# Three step flow focusing enables image-based discrimination and sorting of late stage 1 *Haematococcus pluvialis* cells

**DOI:** 10.1371/journal.pone.0249192

**Published:** 2021-03-29

**Authors:** Daniel Kraus, Andreas Kleiber, Enrico Ehrhardt, Matthias Leifheit, Peter Horbert, Matthias Urban, Nils Gleichmann, Günter Mayer, Jürgen Popp, Thomas Henkel

**Affiliations:** 1 Leibniz Institute of Photonic Technology, Jena, Germany; 2 Gesellschaft zur Förderung von Medizin-, Bio- und Umwelttechnologien e. V. (GMBU), Halle (Saale), Germany; 3 Institute of Physical Chemistry and Abbe Center of Photonics, Friedrich-Schiller-University, Jena, Germany; Bharathidasan University, INDIA

## Abstract

Label-free and gentle separation of cell stages with desired target properties from mixed stage populations are a major research task in modern biotechnological cultivation process and optimization of micro algae. The reported microfluidic sorter system (MSS) allows the subsequent investigation of separated subpopulations. The implementation of a viability preserving MSS is shown for separation of late stage 1 *Haematococcus pluvialis* (HP) cells form a mixed stage population. The MSS combines a three-step flow focusing unit for aligning the cells in single file transportation mode at the center of the microfluidic channel with a pure hydrodynamic sorter structure for cell sorting. Lateral displacement of the cells into one of the two outlet channels is generated by piezo-actuated pump chambers. In-line decision making for sorting is based on a user-definable set of image features and properties. The reported MSS significantly increased the purity of target cells in the sorted population (94%) in comparison to the initial mixed stage population (19%).

## Introduction

Cultivation of microalgae in photo-bioreactors allows the large-scale bioproduction of valuable materials and metabolites for application in health, cosmetics, nutrition and biotechnology. The unicellular green algae *Haematococcus pluvialis* (HP) is one of the most studied organisms in the field of biotechnologically used micro algae [1]. HP is known for its ability to produce great amounts of the natural keto-carotenoid astaxanthin which is accumulated in intracellular lipid vesicle bodies [2]. Up to now the most relevant metabolite is astaxanthin, which is well known as the reddish color of salmon meat. Astaxanthin has antioxidant and radical scavenger activities and is therefore used in medicine and as an additive in sustainable nutrition. This leads to a strong motivation and ongoing demands for the bioproduction of astaxanthin in micro algae.

The biotechnological cultivation of the HP cells proceeds in two major stages. Stage 1 (green HP cells) is the proliferation stage of HP cells and the increasing of biomass. In stage 2 (red HP cells) the HP cells produce astaxanthin by external stress factors. Strain optimization and fermentation procedures are challenged by the complex life cycle of HP, which starts with flagellated early stage 1 embryo cells. In the next fermentation step the cells transform to non-flagellated late stage 1 and start the production of carotenoids. These late stage 1 HP cells are the target cells for the sorting experiments and future investigations. Crucial parameters, that control the life cycle of HP cells were nitrogen starvation as well as high intensity light-stress [3–5]. Nevertheless, there are a lot of other stimuli described in literature that also lead to the induction of the astaxanthin biosynthesis (temperature, salt concentration and osmotic pressure) [4]. Following to the application of inductive stimuli (light, chemical stress) vegetative HP cells transform to dark red cyst cells, which accumulate astaxanthin. In contrast, when cells were continuously exposed to vegetative growth conditions (sufficient amount of nutrients and moderate light intensities) cell division takes place and any single cell is able to go through different multicellular stages from which new embryonical stage 1 cells can be released.

For the optimization of cultivation strategies detailed knowledge on the control mechanism, triggering the carotenoid production is requested. The induction of carotenoid biosynthesis is taking place when specific stimuli were applied to the cells. The modification process and how the stimulus occurs is still not fully investigated and understood. The cells undergo metabolic changes that result in their transformation to late stage 1 cells. Today, numerous metabolic pathways, gene activities and intracellular communications inside the micro algae that were triggered by different cultivation stimuli have not yet been fully decoded and described [6]. For investigation of these mechanism by functional genome analysis, metabolic profiling and proteome analysis pure collections of HP cells of a given sub type need to be isolated from mixed stage population. Cell sorting can be employed for this task. However, HP cells are damaged when exposed to mechanical or shear stress. In this case ruptures in the alginate shell are induced followed by outflow of the cytosol. Therefore, the cell sorting should be realized with low mechanical and shear stress. In the reported work this is implemented utilizing a pure microfluidic sorting concept which is operated at strictly laminar flow conditions at low Reynolds numbers of Re < 10 [7]. This sorting concept can be implemented for image-based sorting utilizing microfluidic devices. Image-based sorting has the advantage of providing the required information about the internal structure and spatial distribution of cellular components.

The reproducible separation of cells with desired target properties from mixed stage populations became a major task in modern biotechnology, drug discovery, diagnostics, cell biology and individualized therapy. Dependent on requirements of purity, throughput, cell population, size and density as well as applied readout and decision-making techniques a multitude cell separation approaches have been developed and put into practice. Also, the preservation of viability of the cells during the sorting process must be considered. One major group of implementations utilizes selective cell binding to functionalized substrate surfaces based on binding interactions between the cell surface and the substrate. After removing unbound cells by washing, the target cells can be released and collected for subsequent processing [8]. Other approaches utilize continuous flow of a cell suspension in combination with single cell analysis, decision making and collection of selected cells into different reservoirs [9, 10]. Right now, conventional fluorescence-activated cell sorting (FACS) with attached sorter functionality and droplet-based sorting of cells, encapsulated in droplets are widely in use. First microfluidic sorter systems became available on the market [11].

Conventional FACS allows high-speed sorting of cells dependent on their optical properties. This method is the gold standard in high-throughput cell sorting for this purpose. Therefore, the overall magnitude of fluorescence and light scattering signals of each cell are measured and used for decision making. Spatial distribution of biomarkers and internal cell structures cannot resolve by this method. These systems are operating at high fluid velocities of ~1 m/s. The used capillaries have typically a diameter of 50 to 200 μm. As a result, the cells are exposed to a high shear stress, which is not compatible with the desired separation task.

Digital microfluidics combined with image-based droplet sorting has been recommended as an alternative for this purpose by many researchers [12–15]. In these approaches, the target cells are encapsulated into droplets for subsequent sorting. Electrical field interactions with the droplets can be utilized for guiding selected droplets to different outlets of the system at actuation rates up to 450 events/second [16]. However, due to the droplet internal circulation the z-position of a single cell inside a droplet is indefinite which causes problems in microscopic imaging due to the limited focal depth of the imaging system in relation to the droplet height. Specialized optical arrangements with tilted focal planes were reported to overcome these limitations [17]. Alternatively, the cell suspension can be directly fed into a flow cell for separation. Therefore, cells pass the flow cell within the focal plane of the imaging system. This can be realized by acoustophoretic,[18–22] dielectrophoretic,[23–26] hydrodynamic,[27–29] optical[30–32] or advection-based[33–36] flow focusing methods. Alternatively, holographic imaging can be used for the 3D morphology characterization of the cells in microfluidic flow [37]. Summarizing, a wide spectrum of separation methods is reported for the implementation of the specific separation task. The combination with image-based sorting is feasible for a subset of them.

Recently, Kleiber *et al*. (2020) reported the three-step hydrodynamic flow focusing of cells for applications in imaging flow cytometry, which operates at low Re <10 and self-alignment of all cells within the focal plane of the imaging system [38, 39]. Combined with a subsequent microfluidic sorter unit the resulting system match the target use case.

In this work we report the implementation of a viability preserving microfluidic sorter system for label-free separation of late stage 1 HP cells. The microfluidic sorter chip combines a three-step flow focusing unit for aligning all cells in single file transportation mode at the center of the microfluidic channel with a pure hydrodynamic sorter structure for binary sorting. Lateral displacement of the cells into one of the two outlet channels is generated by piezo-actuated pump chambers. In-line decision making for sorting is based on a user-definable set of image features and properties. With this approach late stage 1 HP cells can be clearly separated and collected from mixed stage cell population.

## Materials and methods

### Buffers and sample preparation

For the sorting experiments, we used a *Haematococcus pluvialis* strain SAG34-1b that was obtained from the culture collection of algae at Georg August University (Göttingen, Germany). For sorting experiments the cells were previously cultivated in 500 ml glass vessels under the following cultivation parameters. As media an artificial growth media 1xBG-11 without sodium nitrate was used. Illumination of the cells was performed by continuously white (2700 K) and blue (450 nm) LED light with an intensity of 800 μmol m^-2^ s^-1^ and 1065 μmol m^-2^ s^-1^, respectively. The initial cell density was about 2*10⁵ cells/ml. The cultivation was performed for 5 days with continuous fumigation with a gas mixture of air and carbon dioxide (1%) under a flow volume of 350 ml/min.

The HP samples were provided in growth media by GMBU e.V. (Halle, Germany). A solution of Polysucrose 400 (20% w/vol) from Sigma-Aldrich (St. Louis, USA) in tab-water was used as sample and run buffer to prevent sedimentation of the sample. In order to separate disturbing filaments, fragments, empty cell envelopes and agglomerating cell clusters in advance, the sample was filtered by a MACS® SmartStrainer filter (pore size 70 μm) from Miltenyi Biotec (Bergisch Gladbach, Germany).

### Microfluidic devices

The Microfluidic chip device has dimension of 16x25 mm and is prepared as a glass/glass two-layer compound BOROFLOAT® 33 from Schott AG (Mainz, Germany), covered with a 400 μm Polydimethylsiloxane (PDMS) membrane for actuating the two pump chambers. The PDMS membrane is attached to the glass surface by plasma bonding [40]. The microfluidic channel height at the sorting area is 260 μm. The glass wafers, which contain the upper and lower channel half are microlithographic produced by anisotropic wet etching with hydrofluoric acid. Pump chambers were integrated by this technology into the upper face of the upper substrate. The vertical inlets for fluid interconnects were generated by ultrasonic drilling. Substrates were fused by anodic bonding utilizing a silicon bond support layer. Optical access for imaging and microscopy is given by the bottom face of the chip having a cover glass thickness of 0.7 mm and refractive index of 1.47.

### Microfluidic chip integration

The microfluidic chip device and 3D printed actor bridge were integrated into a microscopy slide compatible carrier plate. The actor bridge is made of AR-M2 and was printed with the AGILISTA-3200W from KEYENCE Deutschland GmbH (Neu-Isenburg, Germany). The carrier plate provides fluid connectors for attaching the microfluidic chip to the fluid and sample management unit as well as the mounting facilities for the microfluidic chip and the actor bridge. This plate can be utilized on any conventional upright or inverted microscope, equipped with the matching objectives and digital imaging devices. A minimum working distance of the objective is ~2 mm. Microscopy objectives should have a cover glass thickness correction which matches the depth of the channels inside the microfluidic chips. The chip is connected to neMESYS syringe pumps from CETONI GmbH (Korbußen, Germany) by using PTFE-HPLC tubing ID 0.5 mm.

### Optical setup and microfluidic control

The optical setup was a self-made microscope mounted on an optical bench. A 3W 3000K LED from CREE Inc. (Durham, USA) was used as transmission light source. The cartridge with the microfluidic chip was integrated into an XY-stage from Märzhäuser Wetzlar GmbH & Co. KG (Wetzlar, Germany). A microscopy objective from Mitutoyo Corporation (Kawasaki, Japan) with a 10x magnification, numerical aperture (NA) of 0.28 and working distance (WD) of 34 mm was used. The transmitted light of the sample is split into two channels by using a beam splitter. 80% of the divided light is projected onto the sorting channel. The remaining 20% of the light is directed into the validation channel. In the sorting channel is in addition a narrow bandpass filter FL543.5–10 (CWL = 543.5 ± 2 nm, FWHM = 10 ± 2 nm) from Thorlabs Inc. (Newton, USA) integrated. Two CMOS cameras were used to take the measurement images. The sorter camera was a Manta G223B monochrome camera (2.2 Megapixel) from Allied Vision Technologies GmbH (Stadtroda, Germany). An UI-3370SE from IDS Imaging Development Systems GmbH (Obersulm, Germany) color camera (5 Megapixel) was used for the observation of the sorting area.

### Validation of the sorting quality

To determine the cell concentration of the start population and the two output populations (sorted and waste) a 10 μl Cell Counting Slide from EVETM NanoEnTek (Seoul, Korea) was used. Two counting chambers were counted for each of the start and waste populations. For the sorted populations, six counting chambers were counted due to the low cell concentration. Cells were assigned to late stage 1, if the following criteria are fulfilled: red lipid vesicles in center of the cell, presence of chloroplast, size of 14 ± 3 μm, visible and intact alginate shell and optional flagella. All these features can be easily recognized which leads to a low misclassification rate below 10%.

### Sorter software and decision making

The sorter image processing software is implemented in Java utilizing the OpenCV digital image processing toolkit. The software continuously captures images from a selectable ROI inside the inflow channel of sorter area, detects particles passing the ROI and measures their features, positions and velocities. The utilized sorter camera (AVT-Manta G223B) operates during the sorting process with a ROI size of 300 x 100 pixels and frame rate up to 600 FPS. The ROI covers approx. 1/3 of the channel height. The particles are only detected and calculated when they pass the ROI. Particles that pass above or below the ROI are not detected and go straight to waste channel by default. The following features are utilized for sorter control: Position and velocity of the particles are required for the calculation of the correct timing for triggering the sorter pulse. This trigger releases the TTL signal, when the particle passes the center position of the cross junction inside the sorter area. It is delivered to the sorter microcontroller which generates the power pulses for operating the two piezo actors. Therefore, the software needs to know the direction of the channel in the captured images as the optimum target position for actuation in relation to the position and size of the ROI. These parameters are set in the graphical user interface (GUI) of the software. Additionally, the geometrical features of the particles as well as upper and lower thresholds for decision making can be selected and combined by logical operators. The calculated features can be combined, to separate particles with defined properties.

### Microcontrollers and electronics

We used a self-built microcontroller and Java software to adjust the pulse pattern. For the stroke generation we used mounted piezo actuators P-883.11 form PI Ceramic GmbH (Lederhose, Germany). In combination with the microcontroller we are able to define the deflection, the length as well as the duration of the stroke. For the sorting experiments we used a bias voltage of 1 V for both piezo actors. As working voltage 32 V were used for each direction. One of the two piezo was operated reverse, so that one piezo push and the other pulls at the same time. A full stroke cycle is divided into 5 individual periods. The delay time of the impulse was set to a minimum of 1 ms. The rising edge was set to 50 ms. The plateau phase was set to 200 ms. The falling edge was set to 100 ms, so that the recoil of the stroke does not pull back the sorted particle.

## Results and discussion

### HP cell properties and discrimination between cell stages

**Fig 1:**
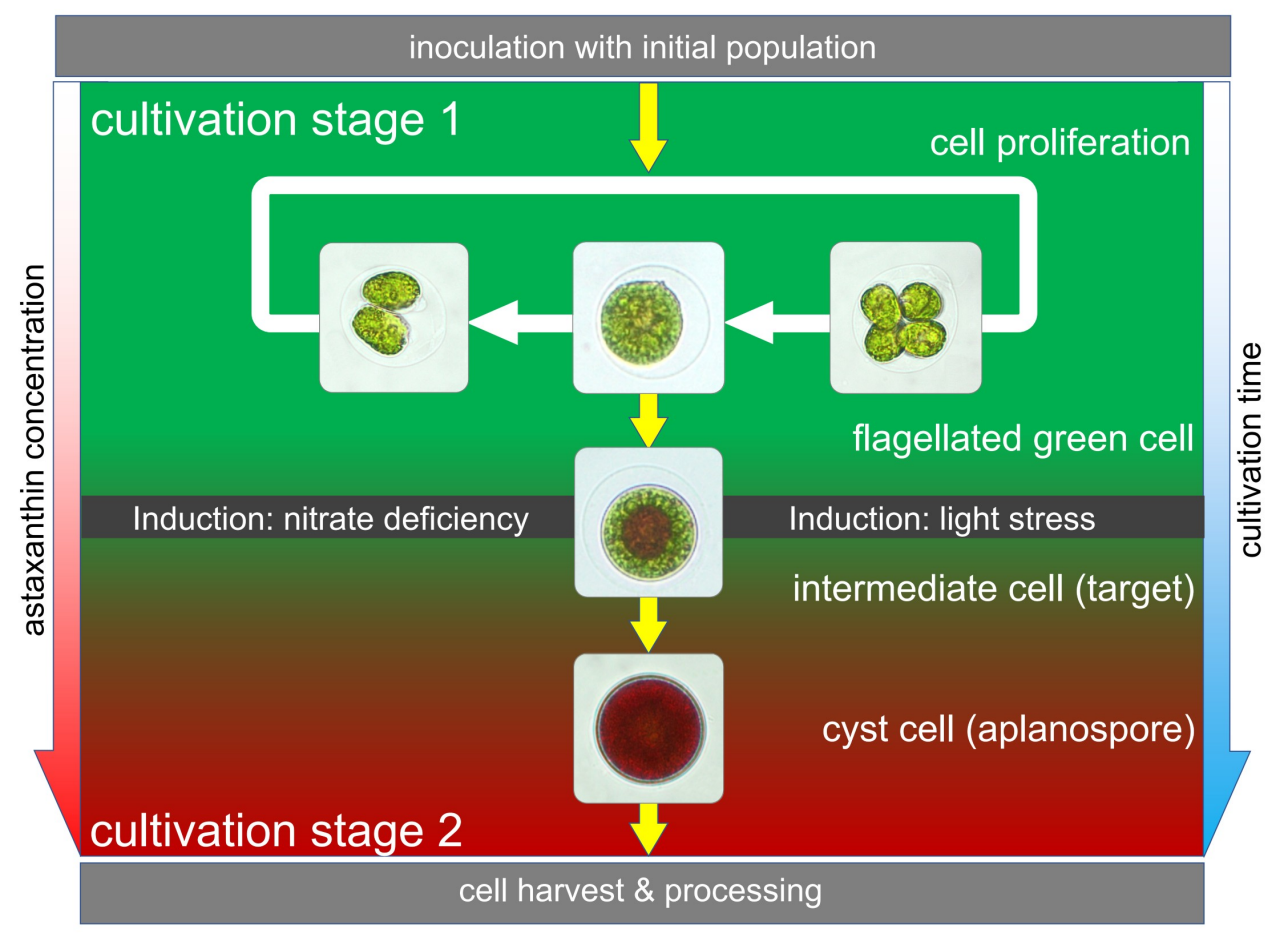
Life cycle of the unicellular microalgae *Haematococcus pluvialis*. Cultivation stage 1: Bipolar flagellated and motile zoospores with high amounts of chlorophyll. Reproduction takes place in this stage by asexual cell proliferation. Induction: Astaxanthin biosynthesis is triggered in the cells by application of high intensity light stress and nitrate starvation. Target cells in the late stage 1 start producing astaxanthin and partially lose their flagella. Cultivation stage 2: Continuous application of inductive stimuli lead to the transformation of green reproductive and motile zoospores to non-motile red aplanospores with high intracellular amounts of astaxanthin and a thick multilayered cell wall.

Geometrical and spectral properties of HP cell types and their relevance for biotechnological fermentation are described in this subsection. Furthermore, spectroscopic and image-based features of the cells are used to differentiate between the cell types for image-based cell sorting. Batch fermentation of HP cells is the standard method for production of astaxanthin in industrial scale. The fermentation process itself is a two-stage process, whereas the first stage is described as the green and the second stage as the red stage (Fig 1). During the green stage the cell proliferation and biomass formation is forced [5, 41]. To maintain the HP cells within the green stage (stage 1) it is necessary to ensure optimal growth conditions that are influenced by different parameters like illumination, media composition, temperature, salinity and others. During the reproductive stage 1, the HP cells are bipolar flagellated, possess a loose cell membrane, and have a cell diameter between 10–20 μm. The color of the HP cells is mainly influenced by their high amounts of chlorophyll.

After the generation of biomass in the green stage, the HP cells are transferred to the red stage (stage 2) of cultivation, where the production of astaxanthin is triggered by external stress factors such as high intensity light-stress and nitrate starvation [41–43]. Following to that the HP cells undergo a sequence of morphological changes which finally end up in the development of red cyst cells with a thick multilayered cell wall and a diameter between 15–30 μm. During the transformation from the stage 1 to stage 2 there are a lot of intermediate stages that can be differentiated by microscopic analysis.

In cultivation stage 2 the color of the cells is mainly determined by the high amounts of the produced natural astaxanthin and its esterified forms which where accumulate within the intracellular lipid vesicle bodies. It is not possible to define directly which of the changes might happen first during the process. The transformation is taking place in the order, that the cells first lose their flagella and build a thick cell wall. Afterwards, the biosynthesis of astaxanthin is induced and the cells become red. The accumulation of astaxanthin starts in the late stage 1 at the center of the cells so that color changes are first visible in the center. Since the accumulation takes place in mobile lipid vesicle bodies, it can be observed that the coloration spreads from the center to the periphery of the cell interior until the whole cyst turned red. It can also be observed that the cells become red even when keeping their flagella and are motile or lose their flagella and stay in a green cyst stage. The clarification of the mechanisms and molecular pathways, which trigger the transition from stage 1 HP cells to astaxanthin producing HP cells is not been fully decoded and described. The key stage for this investigation is the late stage 1 where the formation and accumulation of carotenoids starts.

**Fig 2:**
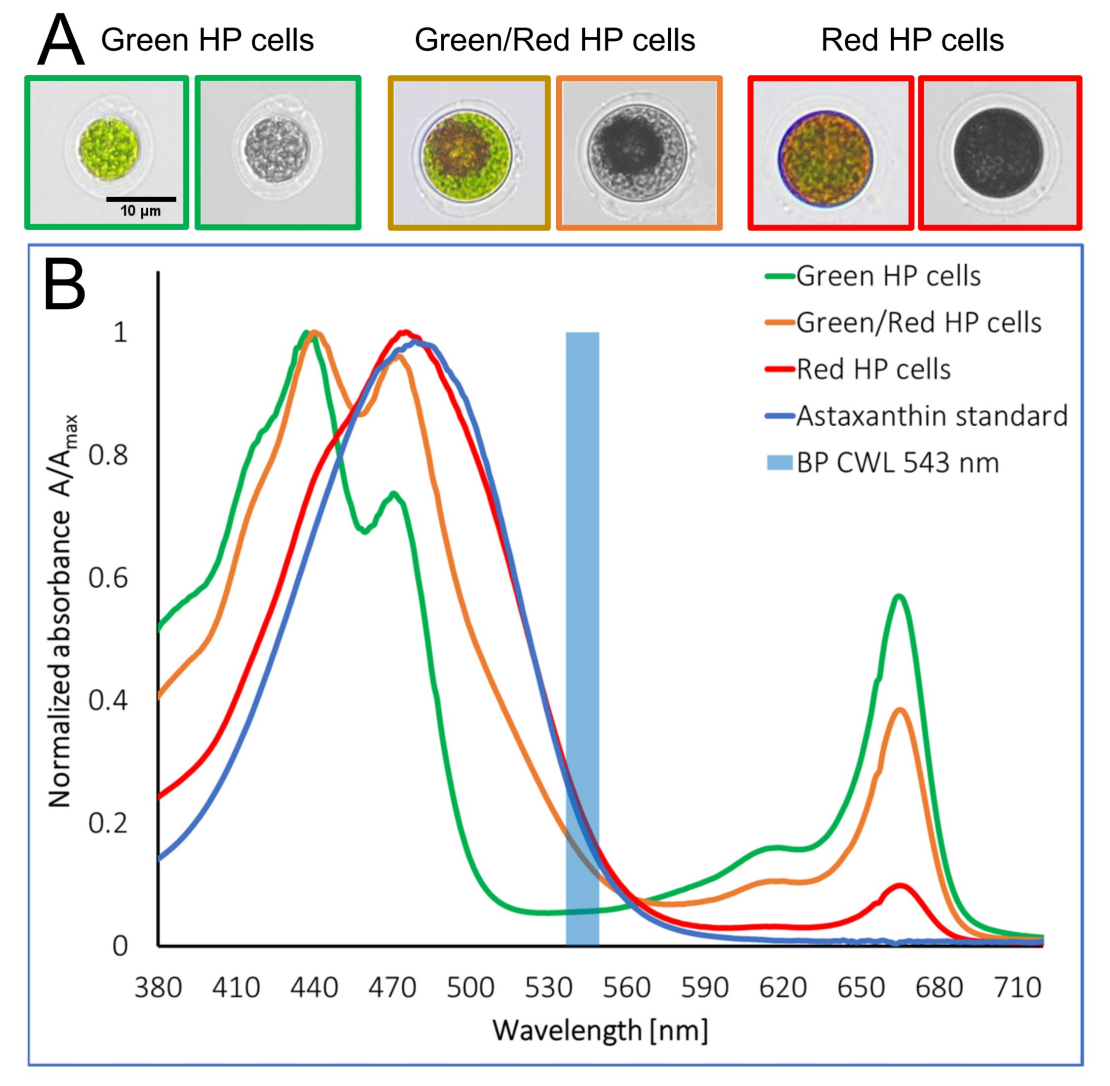
Spectral properties of HP cell lysates from different micro algae sub types. A) RGB image (left) and monochrome image at spectral band 538–548 nm (right) of three relevant micro algae sub types. B) Spectral properties of cell lysates (diluted in Dichloromethane:Methanol (25:75 v/v) green, green/red and red algae as well as pure Astaxanthin as reference. The blue marked band between 538–548 nm is used for imaging. All other bands are blocked by the bandpass filter.

Molecular composition of the HP cells in their different stages is reflected in their microscopic images as well as in the spectral properties of their cell lysates (Fig 2). The lysate spectra of green HP cells are dominated by the absorbance characteristics of chlorophylls with strong structured absorbance bands in the spectral ranges of 400 to 500 nm and 630 to 690 nm. The absorbance of astaxanthin and carotenoids overlaps with the chlorophyll absorbance, but carotenoids can be spectrally separated from chlorophylls within the wavelength range between 530 and 550 nm, where the chlorophylls are transparent. Consequently, the carotenoid content in lysates of green/red and red HP cells can be exclusively measured in the spectral range between 530 and 550 nm. In the lysates of red HP cells with high astaxanthin content the spectra are dominated by astaxanthin, structured chlorophyll bands become invisible in the range between 400 and 500 nm, but the presence of chlorophyll is still visible in the spectral region between 630 and 690 nm. In green/red HP cell lysates the presence of chlorophylls becomes visible for both spectral ranges 400–500 nm and 630–690 nm while the carotenoid content is represented by the absorbance at 530–550 nm.

In the recorded images the situation is more complex, because the absorbance of the molecular components is superposed with the phase contrast effects of the HP cell compartments. This is depicted in the upper part of Fig 2, where RGB images are compared with the monochrome images for the spectral range between 530 and 550 nm. For the monochrome images the contained image information is reduced to two major components–phase contrast and carotenoid absorbance. Grey value distribution and structures reflect the phase contrast. Content and spatial distribution of carotenoids containing lipid vesicle bodies is visible by additionally reduced grey values at their location in the cell images. Target HP cells for the image-based separation are the green/red types where the shape can be identified by the phase contrast signal and the carotenoid content by the decreased grey value level in the center of the cell images which is not observed in the green HP cells. Large vegetative and cyst cells can be identified based on their cross-sectional area in combination with the missing or strong carotenoid absorbance.

### Microfluidic concept & implementation

**Fig 3:**
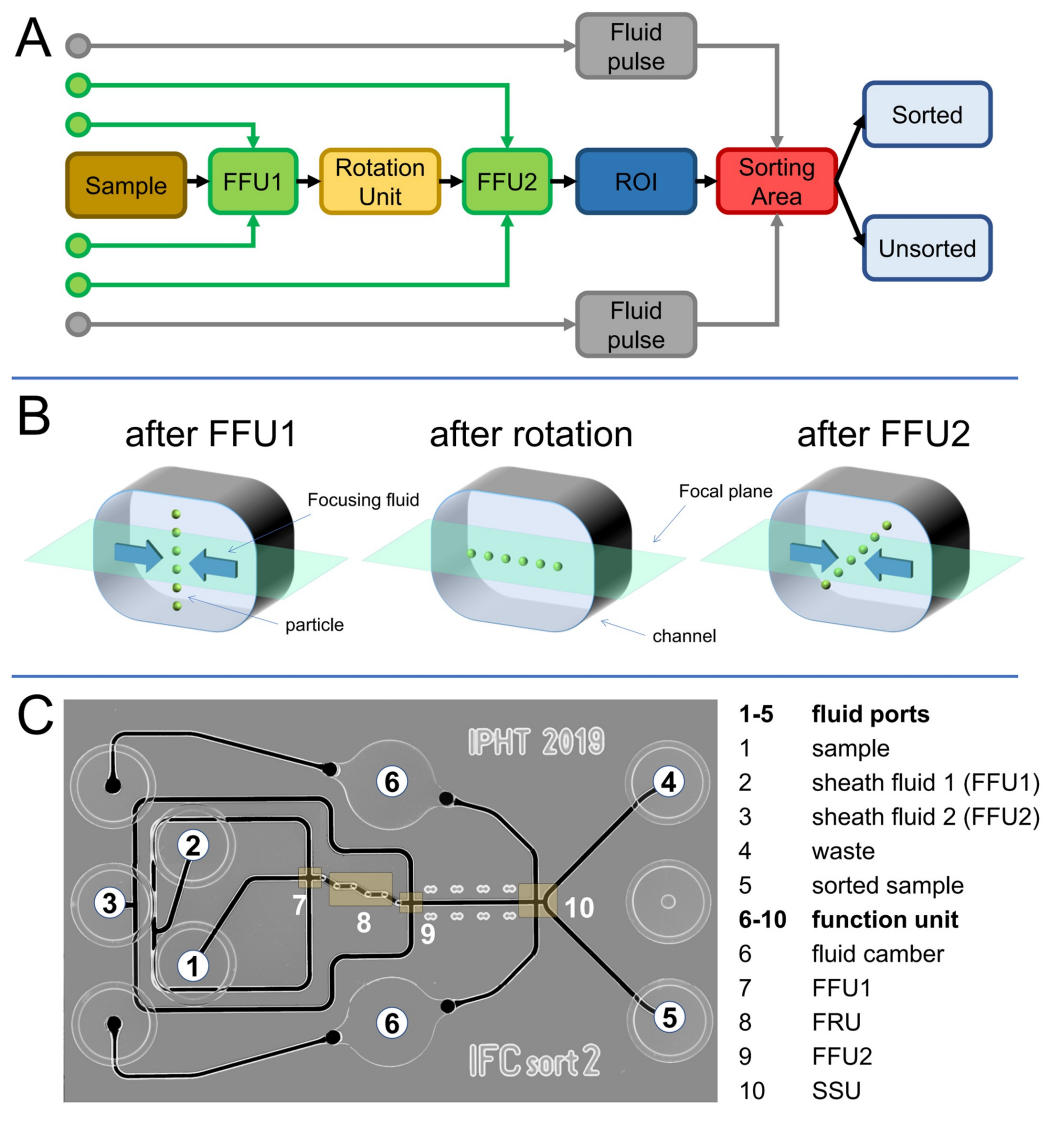
Microfluidic chip concept and functional units. A) Organigram of the implemented functional units of the sorter chip. B) Schematic representations of the three-step flow focusing process. First step the randomly distributed cells are compressed into a vertical lamella at the FFU1. Second step the flow rotation unit (FRU) rotate the vertical lamella for 90 degree into horizontal lamella. In the last step the cells are compressed at FFU2 to single particle file. C) Microscope image of the IFCsort2-Chip.

The sorter device can be put forward as a microfluidic device, which provides the channel structures for fluid transport and particle alignment, pumping chambers for creating fluid pulses as well as actor components for pumping chambers actuation For the microfluidic implementation we follow a modular approach, comprising a passive microfluidic sorter chip with channels and pump chambers at standardized positions (Fig 3). In addition, a 3D printed actor bridge, which generates the stroke for actuating the pump chambers is used. The microfluidic sorter chip itself as the variable part can be easily exchanged while the actor component becomes a permanent part of the system infrastructure. The definition of this chip as the “variable” part allows the utilization of application specific chips, optimized for different particle sizes and application specific, on-chip pre-processing functions on the same sorter system infrastructure as long as the chip geometry, fluid port position and actor chamber positions comply with the specified standards.

The implemented microfluidic sorter chip comprises two operation units, a three-step hydrodynamic flow focusing unit, which aligns all particles in single file transportation mode along the center axis of the inflow channel into the sorter unit and a subsequent sorter unit, which creates and delivers the cross flow for switching the particles between the two outlet channels for sorting (Fig 3B).

### Flow focusing element

Kleiber *et al*. (2020) descripted the detailed implementation of a 3D-hydrodynamic flow focusing unit for self-alignment of particles in a microfluidic channel [38]. For image-based sorting, all particles must be captured and sharply imaged in focal plane of the imaging system. The 3D-hydrodynamic flow focusing unit of the chip is composed from three elements (Fig 3B). In the first step all particles are compressed by sheath flow into a vertical lamella at the middle of the microfluidic channel. In the next step all particles are rotated 90 degrees in the subsequent flow rotation unit into a horizontal orientation lamella at the center height of the microfluidic channel [38, 39]. In the third step a lateral sheath flow is utilized to compress this 2D particle lamella into a single file transportation mode. At the end, all particles reach the sorter unit along the channel axis at a uniform z-position within the focal plane of the imaging system.

### Sorter element

**Fig 4:**
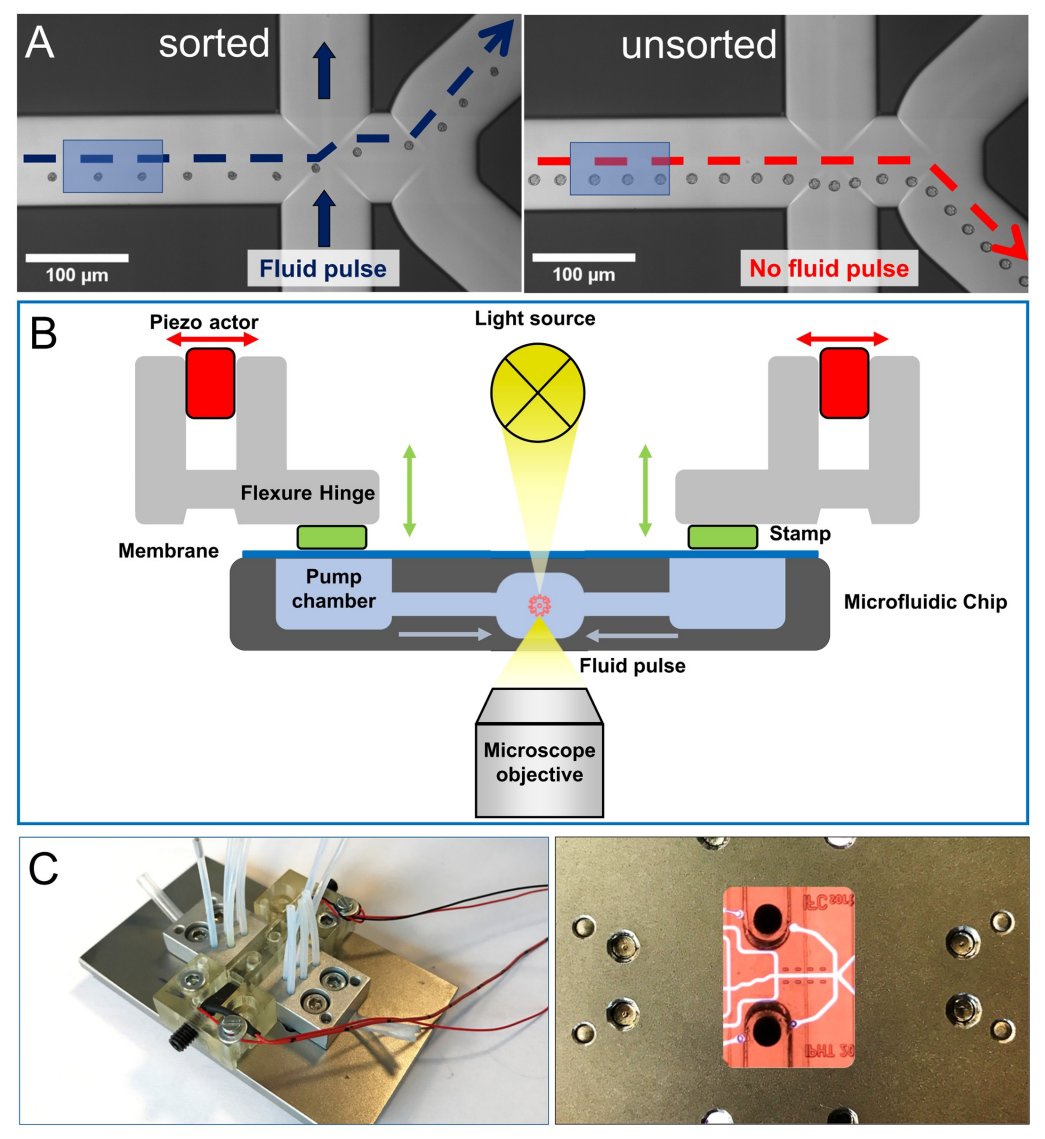
Microfluidic sorting principle and actuator bridge integration. A) Single particle series while passing through the sorting area—sorted (left) and unsorted (right). All particles with the previously defined properties deflected into the sorting channel (upper channel) by the fluid pulse (left). All particles, which are not displaced by a cross flow fluid pulse leave the system through the default outlet (lower channel) (right). The blue rectangles are highlighting the region of interest (ROI) used for in-line image acquisition, digital image analysis and decision making for sorting. The decision point for sorting is precise matched within the time window from leaving the ROI to arriving at the sorting area. B) Cross flow fluid pulses are generated by a pump chamber, covered with a 400 μm thick PDMS membrane. The stroke is created by piezo actors and transferred to the pump chamber membrane via a stamp using a flexure hinge. C) microfluidic chip device built-in a microscopy slide compatible plate covered with 3D printed actor bridge and integrated piezo actors (left). Top view of the optical accessibility for the illumination and imaging system (right).

The sorter unit is composed from a cross junction and a subsequent Y junction. The particle displacement for guiding the particles to different branches of the Y-junction is generated by cross-flow fluid pulses at the cross-junction, which deflects the particles, arriving at the center of the sorting inflow channel towards the left or right side of the microfluidic channel (Fig 4A). Cross-flow is created on-chip by piezo-actuated pump chambers (Fig 4B). Particles can be guided into outlet channels of the downstream Y junction assuming, that the outflow rates in each outlet channel are constant over time. Managing and preserving this situation is complex in terms of control, if both outlets are connected to environmental pressure. Differences or fluctuations in the hydrodynamic resistivity of the outlet branches–e.g. due to an air bubble inside one of them would disturb the hydrodynamic equivalence of the channels. This is solved by using a syringe pump system with constant outflow rate at one of the outlets. The remaining outlet is connected to a given pressure, typically the environmental pressure. The outlet flow rate can be adjusted in a way, that all non-actuated particles are guided to the default outlet (waste). Actuated particles are shifted to the sorted outlet. Under these conditions only particles, which retrieved are actuated. So, we can regulate actuation frequency to the frequency of hit-particles, matching the definable properties for retrieval.

The sorter actor is implemented as a 3D printed actor bridge for mounting on top of the chip (Fig 4C). Two actor stamps are located at the positions of the pump chambers of the chip. Stroke is individually generated for each stamp by piezo elements and transferred to the actor stamps using flexure hinges. A unidirectional cross flow is generated on demand by opposite z-movement of the stamps, actuating the membranes. However, after each pulse actuation the stamps return to the starting position. To accomplish this a full stroke cycle includes a forward stroke, a hold period and a reset stroke.

**Fig 5:**
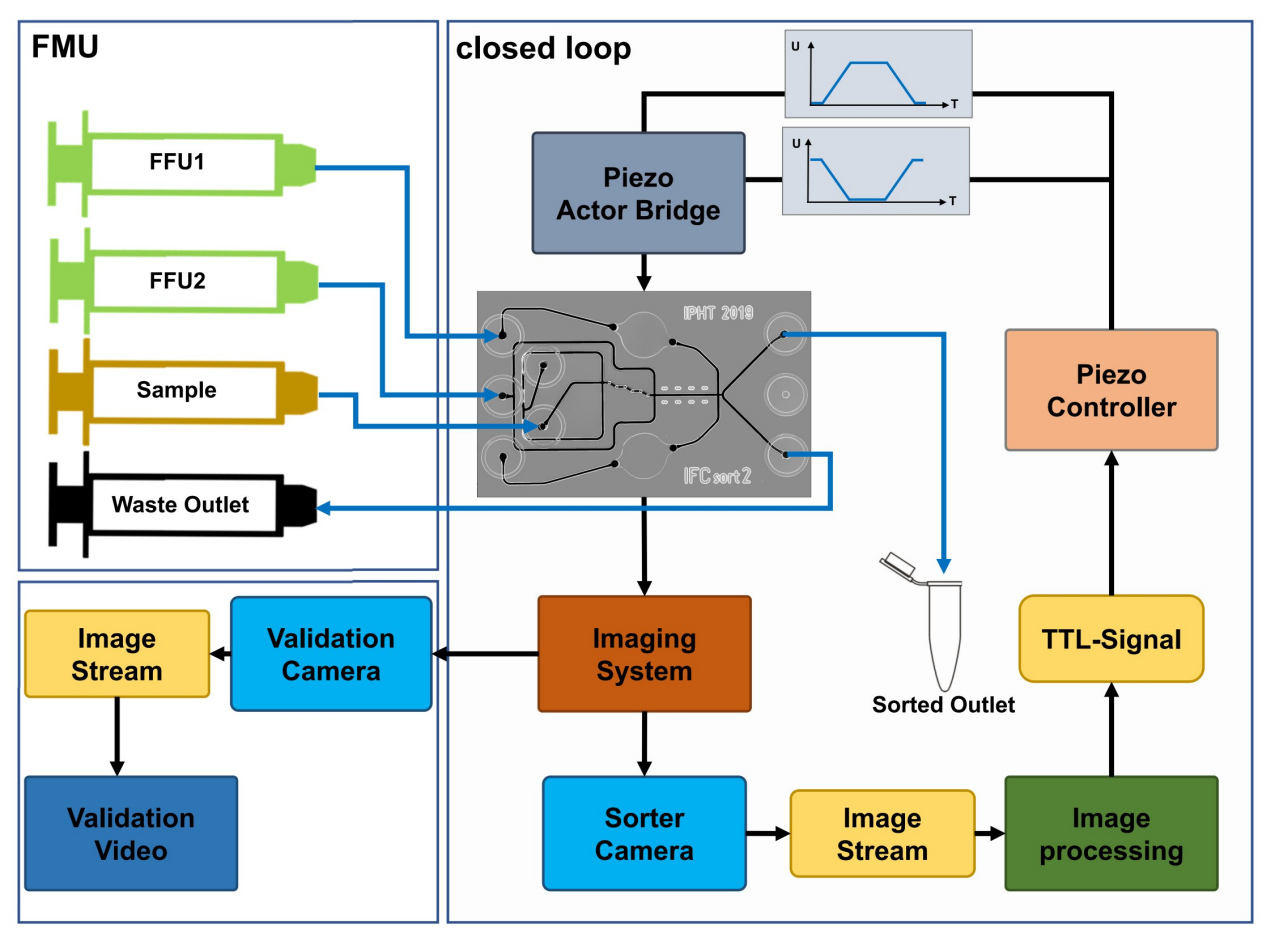
System and control diagram for the sorter infrastructure. Fluid management is provided by the FMU. Sorted particles are collected at the sorter outlet. Two CMOS cameras are implemented into the system. Sorter camera, which images the ROI for sorting and delivers them to the image-based sorting process chain. Validation images of the sorter chip are continuously recorded for independent monitoring and evaluation of the sorting process. Blue connectors indicate tubing’s for fluid transport, black connectors indicate electrical connections for date transfer and power lines.

The network diagram of the sorter system is given in Fig 5. The fluid management unit (FMU) delivers the fluids to the sorter chip. For operating the chip device, the sample flow, the sheath flows at FFU1 and FFU2 as the outflow through the default sorter outlet are controlled by this unit. The imaging system is implemented as a dual head microscope with two complementary metal-oxide-semiconductor (CMOS) cameras. The validation camera acquires a video stream of the sorting area for process monitoring or for subsequent analysis and validation of the sorting process. The sorter camera provides an image stream of the measured ROI which is used as the input data for the image-based sorting process. This image stream is connected to the processing software, which analyzes the image sequence, performs the decision making for sorting and provide a Transistor-Transistor Logic (TTL) signal. This event is captured and converted by the piezo controller into power pulses for operating the two piezo elements in the actor bridge, which are generating the required cross flow for guiding the particle into the sorted outlet of the chip.

### HP sorting and results

**Fig 6:**
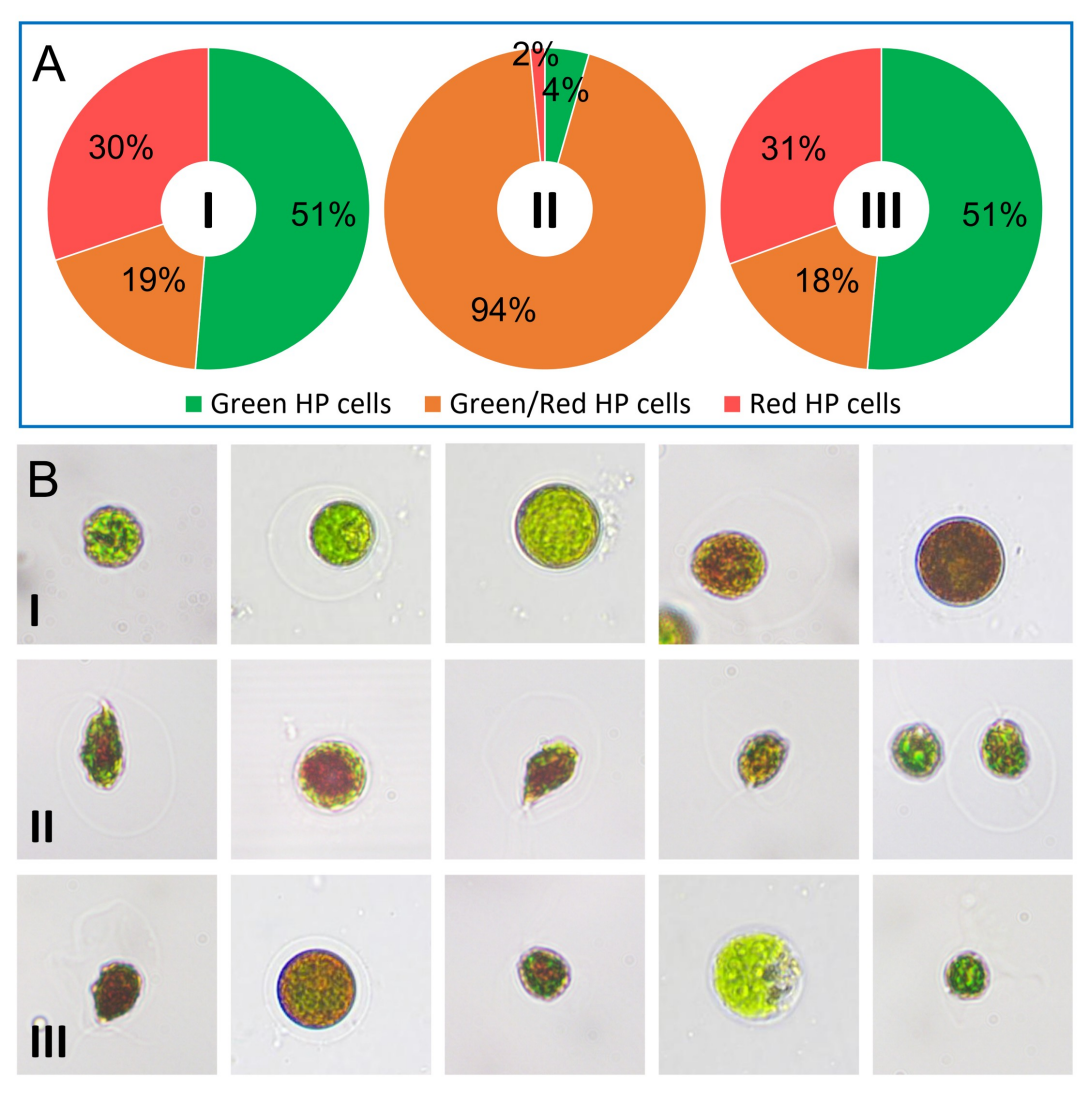
Composition of cells in the different fractions of the sorting process. A) Percentage distribution of the relevant cell type fractions I) Initial population II) Sorted population III) Waste population B) Microscopy images of the three fractions. Sorted population with the given criteria for sorted cells in terms of size, an intact alginate shell, a visible flagellum and the presence of initially formed carotenoids in the center of the cells.

To demonstrate the usability of our microfluidic sorter system, we used a mixed stage population of HP cells with different cell stages. The cell stages present in the mixed stage population were previously categorized into 3 major stages. This initial used population contained 51% stage 1 (green), 19% late stage 1 (green/red) and 30% stage 2 (red) HP cells (Fig 6AI). Late stage 1 cells are the target cells for the subsequent investigations and should therefore be separated. Target HP cells are in a single cell stage, covered with an alginate shell and have a flagella. The inner volume of the cell contains chloroplasts and in the center region an initial fraction of reddish lipid vesicle bodies containing the carotenoids (Fig 6BII). For the experiments the initial cell population is suspended in the sample buffer. This buffer is optimized to match the density of the HP cells, which avoids sedimentation in the feed lines. The same buffer is also used for the sheath fluids at FFU1 and FFU2. A more detailed description of the HP cells as well as the buffer can be found in the section “Materials and Methods”.

The reported application is not suited for high throughput but for pure and gentle separation of specific cell type. With this method of separation, the HP cells preserve their vitality and can be collected and further investigated. The throughput depends on the sample and the image processing speed for decision making. For sorting, the cells must pass the sorting point individually and one by one, with sufficient distance between the cells. Samples that tend to agglomerate must therefore be present in an appropriate low concentration, otherwise cell clusters will be formed. If the distance between two consecutive cells is not sufficiently large, it can happen that both cells are deflected into the sorting channel. This leads to an incorrect sorting. Furthermore, it is possible that the cells are not perfectly struck by the fluid pulse and thus not enough displacement to deflect the cell into the sorting channel.

The sorting criteria for the target HP cells were narrowly selected so that only late stage 1 HP cells were separated. For spectral discrimination between chlorophylls and carotenoids we used a narrow bandpass filter with a CWL = 543.5 ± 5 nm. The different HP cells can be distinguished by the corresponding grey value and their size. As target parameters a mean diameter between 11.5–16.5 μm and a mean gray value between 60–75 was determined. The combination of these parameters excludes cell fragments, empty envelops, cells that are too small or even to large, as well as cells with excessive chlorophyll or carotenoid content. Therefore, a majority of the cells in the sample do not match the target parameters.

In the experiment 1.3 cells per second passed the ROI. A total of 6990 cells have passed the ROI in microfluidic channel during the experiment. For independent validation and evaluation, a video file was recorded from the experiment. All cell populations (initial (I), sorted (II) and waste (III)) were subjected to microscopic analysis, manual classification and counting (Fig 6). Counting and classification criteria are described in Materials and Methods. A total of 695 cells could be microscopic counted in the sorted population (II) of which 654 were late stage 1 HP cells (target cells). This corresponds to a purity of 94%. The vitality of the cells was observed and confirmed. The proportion of incorrectly sorted cells comes mainly from the cells dragged along during the sorting pulse.

## Conclusion

A microfluidic sorter system for image-based cell separation has been successfully developed and applied. The setup allows the separation of a defined cell type (HP cells of late stage 1) with a purity of 94% from a mixed stage cell population. The early and late stage 1 have similar morphology. They only differ in their content of chlorophylls and carotenoids. Viability of the separated cell population has been confirmed by microscopy. The cell types became separated as pure and viable cell collections for subsequent scientific research on intracellular changes at the level of metabolomics, transcriptomics and proteomics that occur during the described transformation processes.

## Supporting information

S1 Fig Microscopic images of the 3 differnent HP cell stages. Left: stage 1 (green HP cells) with flagellum and alginate envelope. Middle: late stage 1 (green/red HP cells) with flagellum, alginate envelope and start of astaxanthin production. Right: stage 2 (red HP cells) cytosis form and with astaxanthin concentration.
(TIF)

S2 Fig Comparison of RGB and bandpass filter microscope images of the HP cells. The upper part shows the different HP cell stages as RGB image. The lower part shows the same HP cells by using the narrow band pass filter CWL = 543.5 ± 5 nm. The filter allows to visualize specific internal structures of the cells.
(TIF)

S1 Video Focusing of the HP cells in vertical lamella. The video gives an insight about the cell concentration at the entrance of the microfluidic chip. It also shows the flow focusing unit 1 (FFU1) and how the cells are pressed through the lateral fluids into a vertical lamella before they pass into the flow rotation unit (FRU).
(MP4)

S2 Video Overview of the sorting principle. This video shows how the sorting principle works in real application. The video was recorded with the validation camera.
(MP4)

## References

[1] Ambati RR, Phang S-M, Ravi S, Aswathanarayana RG. Astaxanthin: sources, extraction, stability, biological activities and its commercial applications—a review. Marine drugs. 2014;12(1):128–52. 10.3390/md12010128 24402174 PMC3917265

[2] Shah M, Mahfuzur R, Liang Y, Cheng JJ, Daroch M. Astaxanthin-producing green microalga Haematococcus pluvialis: from single cell to high value commercial products. Frontiers in plant science. 2016;7:531. 10.3389/fpls.2016.00531 27200009 PMC4848535

[3] Fábregas J, Dominguez A, Regueiro M, Maseda A, Otero A. Optimization of culture medium for the continuous cultivation of the microalga Haematococcus pluvialis. Applied Microbiology and Biotechnology. 2000;53(5):530–5. 10.1007/s002530051652 10855711

[4] Li J, Zhu D, Niu J, Shen S, Wang G. An economic assessment of astaxanthin production by large scale cultivation of Haematococcus pluvialis. Biotechnology Advances. 2011;29(6):568–74. 10.1016/j.biotechadv.2011.04.001 21497650

[5] Wayama M, Ota S, Matsuura H, Nango N, Hirata A, Kawano S. Three-dimensional ultrastructural study of oil and astaxanthin accumulation during encystment in the green alga Haematococcus pluvialis. PloS one. 2013;8(1):e53618. Epub 2013/01/18. 10.1371/journal.pone.0053618 23326471 PMC3543331

[6] Ota S, Morita A, Ohnuki S, Hirata A, Sekida S, Okuda K, et al. Carotenoid dynamics and lipid droplet containing astaxanthin in response to light in the green alga Haematococcus pluvialis. Scientific reports. 2018;8(1):1–10. 10.1038/s41598-017-17765-5 29618734 PMC5884812

[7] Wuest W. Strömung durch schlitz-und lochblenden bei kleinen Reynolds-zahlen. Ingenieur-Archiv. 1954;22(6):357–67.

[8] Wan Y, Liu Y, Allen PB, Asghar W, Mahmood MA, Tan J, et al. Capture, isolation and release of cancer cells with aptamer-functionalized glass bead array. Lab on a chip. 2012;12(22):4693–701. Epub 2012/09/18. 10.1039/c2lc21251j 22983436 PMC3498495

[9] Gossett DR, Weaver WM, Mach AJ, Hur SC, Tse HT, Lee W, et al. Label-free cell separation and sorting in microfluidic systems. Analytical and bioanalytical chemistry. 2010;397(8):3249–67. Epub 2010/04/27. 10.1007/s00216-010-3721-9 20419490 PMC2911537

[10] Lee W, Tseng P, Di Carlo D. Microtechnology for cell manipulation and sorting: Springer; 2017.

[11] Rajkumar JS, Maddison M, Singh D, COLOMBE P, Spector M, Young MJ. Enrichment of a Retinal Ganglion Cell Population using a Novel Microfluidic Sorting Strategy. Investigative Ophthalmology & Visual Science. 2020;61(7):2521–.

[12] Wu L, Chen P, Dong Y, Feng X, Liu B-F. Encapsulation of single cells on a microfluidic device integrating droplet generation with fluorescence-activated droplet sorting. Biomedical microdevices. 2013;15(3):553–60. 10.1007/s10544-013-9754-z 23404263

[13] Girault M, Kim H, Arakawa H, Matsuura K, Odaka M, Hattori A, et al. An on-chip imaging droplet-sorting system: a real-time shape recognition method to screen target cells in droplets with single cell resolution. Scientific reports. 2017;7:40072. Epub 2017/01/07. 10.1038/srep40072 28059147 PMC5216404

[14] Anagnostidis V, Sherlock B, Metz J, Mair P, Hollfelder F, Gielen F. Deep learning guided image-based droplet sorting for on-demand selection and analysis of single cells and 3D cell cultures. Lab on a chip. 2020;20(5):889–900. Epub 2020/01/29. 10.1039/d0lc00055h . 31989120

[15] Sesen M, Whyte G. Image-Based Single Cell Sorting Automation in Droplet Microfluidics. Scientific reports. 2020;10(1). 10.1038/s41598-020-65483-2 32457421 PMC7250914

[16] Caen O, Schütz S, Jammalamadaka MS, Vrignon J, Nizard P, Schneider TM, et al. High-throughput multiplexed fluorescence-activated droplet sorting. Microsystems & nanoengineering. 2018;4(1):1–10. 10.1038/s41378-018-0033-2 31057921 PMC6220162

[17] Gorthi SS, Schonbrun E. Phase imaging flow cytometry using a focus-stack collecting microscope. Optics letters. 2012;37(4):707–9. 10.1364/OL.37.000707 22344155

[18] Augustsson P, Magnusson C, Nordin M, Lilja H, Laurell T. Microfluidic, label-free enrichment of prostate cancer cells in blood based on acoustophoresis. Analytical chemistry. 2012;84(18):7954–62. Epub 2012/08/18. 10.1021/ac301723s 22897670 PMC3445767

[19] Yang AH, Soh HT. Acoustophoretic sorting of viable mammalian cells in a microfluidic device. Analytical chemistry. 2012;84(24):10756–62. Epub 2012/11/20. 10.1021/ac3026674 23157478 PMC3677785

[20] Schmid L, Weitz DA, Franke T. Sorting drops and cells with acoustics: acoustic microfluidic fluorescence-activated cell sorter. Lab on a chip. 2014;14(19):3710–8. 10.1039/c4lc00588k 25031157

[21] Wu M, Ozcelik A, Rufo J, Wang Z, Fang R, Huang TJ. Acoustofluidic separation of cells and particles. Microsystems & nanoengineering. 2019;5(1):32. 10.1038/s41378-019-0064-3 31231539 PMC6545324

[22] Olofsson K, Hammarström B, Wiklund M. Acoustic separation of living and dead cells using high density medium. Lab on a chip. 2020;20(11):1981–90. 10.1039/d0lc00175a 32356853

[23] Cheng I-F, Chang H-C, Hou D, Chang H-C. An integrated dielectrophoretic chip for continuous bioparticle filtering, focusing, sorting, trapping, and detecting. Biomicrofluidics. 2007;1(2):021503. 10.1063/1.2723669 PMC2717572 19693376

[24] Li Y, Dalton C, Crabtree HJ, Nilsson G, Kaler KV. Continuous dielectrophoretic cell separation microfluidic device. Lab on a chip. 2007;7(2):239–48. Epub 2007/02/03. 10.1039/b613344d . 17268627

[25] Baret JC, Miller OJ, Taly V, Ryckelynck M, El-Harrak A, Frenz L, et al. Fluorescence-activated droplet sorting (FADS): efficient microfluidic cell sorting based on enzymatic activity. Lab on a chip. 2009;9(13):1850–8. Epub 2009/06/18. 10.1039/b902504a . 19532959

[26] Sciambi A, Abate AR. 10.1039/c4lc01194e . Lab on a chip. 2015;15(1):47–51. Epub 2014/10/30. PubMed Central PMCID: PMC4256106. 25352174 PMC4256106

[27] Chabert M, Viovy JL. Microfluidic high-throughput encapsulation and hydrodynamic self-sorting of single cells. Proceedings of the National Academy of Sciences of the United States of America. 2008;105(9):3191–6. Epub 2008/03/05. 10.1073/pnas.0708321105 18316742 PMC2265149

[28] Karimi A, Yazdi S, Ardekani A. Hydrodynamic mechanisms of cell and particle trapping in microfluidics. Biomicrofluidics. 2013;7(2):021501. 10.1063/1.4799787 24404005 PMC3631262

[29] Sun J, Liu C, Li M, Wang J, Xianyu Y, Hu G, et al. Size-based hydrodynamic rare tumor cell separation in curved microfluidic channels. Biomicrofluidics. 2013;7(1):011802. 10.1063/1.4774311 24396523 PMC3555910

[30] MacDonald MP, Spalding GC, Dholakia K. Microfluidic sorting in an optical lattice. Nature. 2003;426(6965):421–4. 10.1038/nature02144 14647376

[31] Wang X, Chen S, Kong M, Wang Z, Costa KD, Li RA, et al. Enhanced cell sorting and manipulation with combined optical tweezer and microfluidic chip technologies. Lab on a chip. 2011;11(21):3656–62. Epub 2011/09/16. 10.1039/c1lc20653b . 21918752

[32] Peřina J, Ježek J, Pilát Z, Šerý M, Kaňka J, Samek O, et al. Microfluidic systems for optical sorting. 18th Czech-Polish-Slovak Optical Conference on Wave and Quantum Aspects of Contemporary Optics, 86970W. 2012;8697:86970W. 10.1117/12.2008649

[33] Nivedita N, Papautsky I. Continuous separation of blood cells in spiral microfluidic devices. Biomicrofluidics. 2013;7(5):54101. Epub 2014/01/10. 10.1063/1.4819275 24404064 PMC3779264

[34] Schaap A, Dumon J, Toonder Jd. Sorting algal cells by morphology in spiral microchannels using inertial microfluidics. Microfluidics and Nanofluidics. 2016;20(9). 10.1007/s10404-016-1787-1

[35] Syed MS, Rafeie M, Vandamme D, Asadnia M, Henderson R, Taylor RA, et al. Selective separation of microalgae cells using inertial microfluidics. Bioresource technology. 2018;252:91–9. Epub 2018/01/07. 10.1016/j.biortech.2017.12.065 . 29306136

[36] Liu N, Petchakup C, Tay HM, Li KHH, Hou HW. Applications of Microfluidic Systems in Biology and Medicine 2019. 99–150 p.

[37] Memmolo P, Villone M, Merola F, Mugnano M, Miccio L, Maffettone PL, et al. Holographic imaging for 3D cells morphology in microfluidic flow. European Conference on Biomedical Optics. 2019:11076_69.

[38] Kleiber A, Ramoji A, Mayer G, Neugebauer U, Popp J, Henkel T. 3-Step flow focusing enables multidirectional imaging of bioparticles for imaging flow cytometry. Lab on a chip. 2020;20(9):1676–86. 10.1039/d0lc00244e 32282005

[39] Henkel T, Bauer M, Neugebauer U, Popp J. Arrangement for individualized patient blood analysis. Patent. 2015.

[40] Bhattacharya S, Datta A, Berg JM, Gangopadhyay S. Studies on surface wettability of poly (dimethyl) siloxane (PDMS) and glass under oxygen-plasma treatment and correlation with bond strength. Journal of microelectromechanical systems. 2005;14(3):590–7.

[41] Su Y, Wang J, Shi M, Niu X, Yu X, Gao L, et al. Metabolomic and network analysis of astaxanthin-producing Haematococcus pluvialis under various stress conditions. Bioresource technology. 2014;170:522–9. 10.1016/j.biortech.2014.08.018 25164345

[42] Vidhyavathi R, Venkatachalam L, Sarada R, Ravishankar GA. Regulation of carotenoid biosynthetic genes expression and carotenoid accumulation in the green alga Haematococcus pluvialis under nutrient stress conditions. Journal of Experimental Botany. 2008;59(6):1409–18. 10.1093/jxb/ern048 18343887

[43] Saha SK, McHugh E, Hayes J, Moane S, Walsh D, Murray P. Effect of various stress-regulatory factors on biomass and lipid production in microalga Haematococcus pluvialis. Bioresource technology. 2013;128:118–24. 10.1016/j.biortech.2012.10.049 23196231

