# Supplementary figures and images for: Three step flow focusing enables image-based discrimination and sorting of late stage 1 Haematococcus pluvialis cells
Source: PLoS One. 2021 Mar 29;16(3):e0249192. doi: 10.1371/journal.pone.0249192 (PMC8007022; doi:10.1371/journal.pone.0249192)

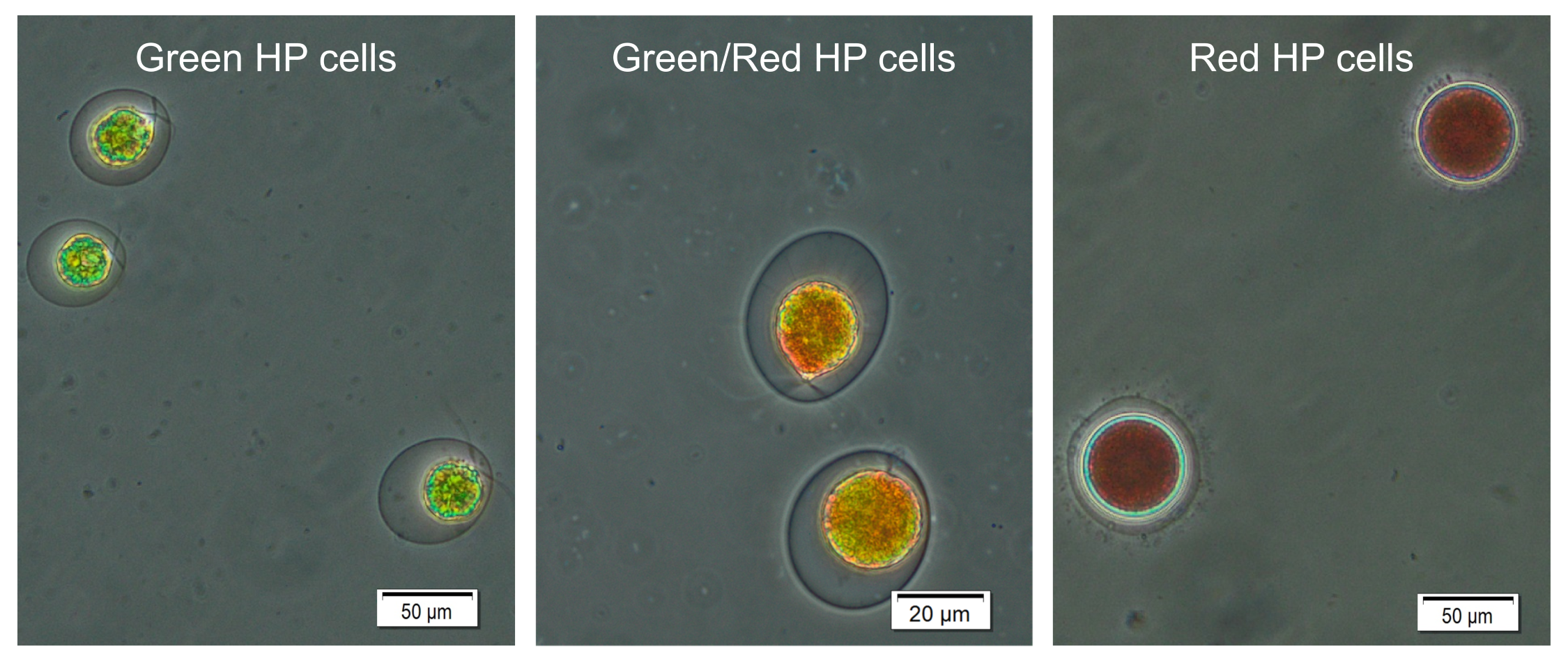

Supplement: S1 Fig — Left: stage 1 (green HP cells) with flagellum and alginate envelope. Middle: late stage 1 (green/red HP cells) with flagellum, alginate envelope and start of astaxanthin production. Right: stage 2 (red HP cells) cytosis form and with astaxanthin concentration. (TIF) [file pone.0249192.s001.tif]

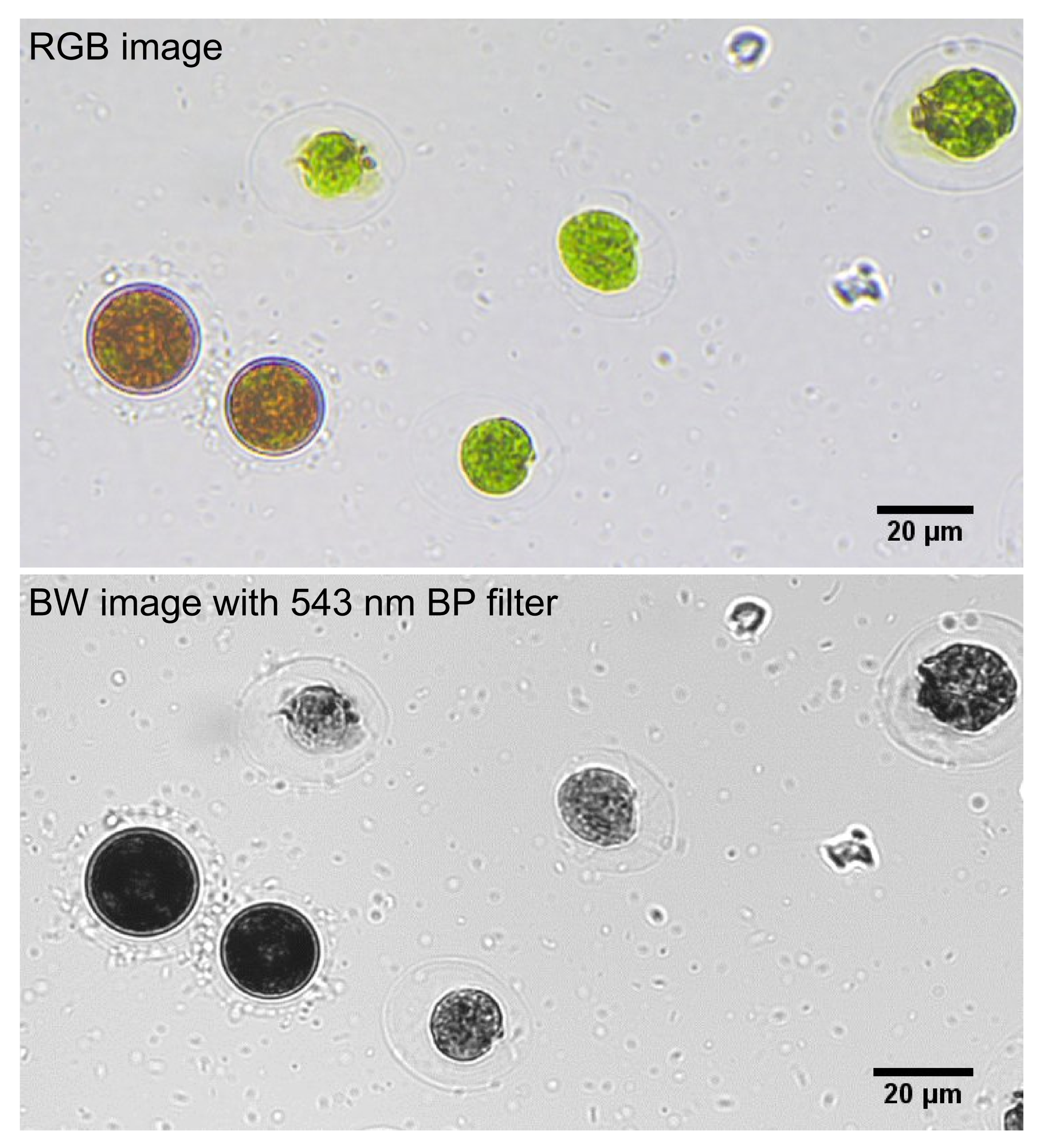

Supplement: S2 Fig — The upper part shows the different HP cell stages as RGB image. The lower part shows the same HP cells by using the narrow band pass filter CWL = 543.5 ± 5 nm. The filter allows to visualize specific internal structures of the cells. (TIF) [file pone.0249192.s002.tif]
